# Machine learning enables non-Gaussian investigation of changes to peripheral nerves related to electrical stimulation

**DOI:** 10.1038/s41598-024-53284-w

**Published:** 2024-02-02

**Authors:** Andres W. Morales, Jinze Du, David J. Warren, Eduardo Fernández-Jover, Gema Martinez-Navarrete, Jean-Marie C. Bouteiller, Douglas C. McCreery, Gianluca Lazzi

**Affiliations:** 1 Department of Biomedical Engineering, University of Southern California, Los Angeles, CA 90089 USA; 2 Department of Electrical Engineering, University of Southern California, Los Angeles, CA 90089 USA; 3 Department of Biomedical Engineering, University of Utah, Salt Lake City, UT 84112 USA; 4 Institute of Bioengineering, Elche and CIBER-BBN, University Miguel Hernandez, Elche, Spain; 5 Huntington Medical Research Institute, Pasadena, CA 91105 USA; 6 Department of Ophthalmology, University of Southern California, Los Angeles, CA 90089 USA; 7 Institute for Technology and Medical Systems (ITEMS), Keck School of Medicine, University of Southern California, Los Angeles, CA 90089 USA

**Keywords:** Neuroscience, Medical research, Neurology, Computer science, Scientific data

## Abstract

Electrical stimulation of the peripheral nervous system (PNS) is becoming increasingly important for the therapeutic treatment of numerous disorders. Thus, as peripheral nerves are increasingly the target of electrical stimulation, it is critical to determine how, and when, electrical stimulation results in anatomical changes in neural tissue. We introduce here a convolutional neural network and support vector machines for cell segmentation and analysis of histological samples of the sciatic nerve of rats stimulated with varying current intensities. We describe the methodologies and present results that highlight the validity of the approach: machine learning enabled highly efficient nerve measurement collection, while multivariate analysis revealed notable changes to nerves’ anatomy, even when subjected to levels of stimulation thought to be safe according to the Shannon current limits.

## Introduction

The peripheral nervous system mediates sensation, motion, and autonomic processes, thus providing a target for neurostimulation to treat many disorders^1–12^. However, to this date, there is limited research into safety guidelines for peripheral nerve stimulation^13–22^. A prominent stimulation guideline, known as the Shannon criterion^23^ (or, as referred to in this paper, the Shannon current limit), was developed from compiling very labor-intensive qualitative measurements of the central nervous system from many expert histologists^24,25^. It has endured as a safety guideline as it provides a quick means of assessing stimulation safety using the easily calculated charge density per phase and charge per phase of the stimulation.

As the usage of peripheral nerve stimulation has increased since the development of the Shannon current limit, so has the number of ways in which we quantify measurements of nerve histology^26–29^. Traditional (i.e., non-automated) methods of collecting these morphometrics are very time-consuming and must be performed by multiple observers. After going through the extensive work of feature extraction, the measurements need analyzing to identify changes and differentiate physical changes to the nerve due to electrical stimulation from changes due to the surgical procedure or immune response to the implanted device. This is complicated by morphometric measurements often having non-Gaussian multi-modal distributions^26–29^. Most statistical analysis methods used to investigate morphological data, to varying degrees, rely on the assumption that the data fits a Gaussian distribution, and there are many techniques for best-fitting data into a Gaussian distribution for the analysis to be valid. However, multimodal distributions completely invalidate those methods and even common summary statistics like central tendency and dispersion. Non-parametric statistics have developed out of a need to analyze this type of data without being constrained to an assumed underlying distribution^30^.

Here we present a novel machine learning-based approach to investigate the impact of electrical stimulation on peripheral nerves. Machine learning (ML), in the form of a convolutional neural network (CNN) and support vector machines (SVM), was used as powerful tools for reducing the burdens of time-consuming image segmentation^31^ as well as complicated non-parametric data analysis^32,33^ to capture morphological and structural changes otherwise difficult to isolate. Our proposed approach reduces labor limitations on the quantity of measurements collected and locations of the nerve where measurements are achievable. And importantly, by reducing the dimensionality of the features extracted, our methods enable observation of structural changes in nerves due to different sources.

## Results

Once the rat sciatic nerves samples (control, sham, and stimulated with cuff electrodes—see “Methods and procedures” Section) were fixed, sliced, and imaged; feature extraction using traditional techniques required, on average, 1 week for each sample. After the CNN was trained, segmentation and post-processing required, on average, 2.5 h per sample; labeling thousands of cells per sample. Automated morphometric and cell-wise structural feature (see “Automated High Throughput Measurements” Section) extraction took, on average, 1.5 h per sample. Automated pixel-wise structural feature extraction (see “Automated High Throughput Measurements” Section) took, on average, 12 h per sample.

As shown in Table 1, SVM regressions were unable to fit purely cell-wise morphometric data (manual or automated measurements) to target values. Combining morphometrics with structural windowed metrics into a larger cell-wise multivariate data set did not improve regression fitting compared to large-windowed metrics alone (surgical regression R^2^ decreased by 0.032 and stimulation regression R^2^ decreased by 0.155). Surprisingly, survivorship bias expected from cell-wise data plays a limited to non-existent role in quality of regression fitting (Comparing cell-wise to pixel-wise large window training vectors: the R^2^ for the surgical regression decreased by only 0.015 and the stimulation regression R^2^ actually increased by 0.03); meaning, cell-wise measurements may be a viable low computational-cost alternative to pixel-wise measurements, despite seeing no benefit from combining with morphometric data.

**Table 1:** Coefficients of determination (R^2^).

Training vectors	Target values	
	Surgery	Shannon
Manual morphometrics	0.227	0.311
Manual fiber density (normal cells only)	0.150	0.392
Manual fiber density (normal, degenerated, & hypomyelinated cells)	0.593	0.536
Automated Morphometrics	0.121	0.149
Automated cell-wise windowed metrics		
Small window	0.504	0.431
Large window	0.709	0.760
Automated morphometrics & cell-wise windowed metrics		
Small window	0.519	0.404
Large window	0.677	0.605
Automated pixel-wise windowed metrics		
Small window	0.525	0.404
Large window	0.724	0.730

Support vector machine regressions were trained on subsets of measurements/features called training vectors. Performance was evaluated by measuring the coefficient of determination between model outputs and sample target values. For surgical regressions, the target value was whether samples had an electrode surgically implanted (1) or not (0). For stimulation regressions, model performance was evaluated by using each samples’ stimulation level (Shannon k) as the target value.

The best fit SVM regressions came from data sets using large windowed structural metrics. This is likely due to how we treat each nerve sample as having homogeneous stimulation target values (Shannon *k*). A more localized measurement or estimation of the electrical stimulation experienced by nerve fibers might enable better regression fitting using the small window measurements and morphometric data, and thus give a more localized measurement of nerve changes specifically related to stimulation.

Fitting regressions to manual density measurements was greatly improved (increased by 0.443 for the surgical regression and 0.144 for the stimulation regression) by including density for rarer damage-related cell classes, degenerated and hypomyelinated cells. Degenerated and hypomyelinated cells were entirely absent from control samples and in the sham samples constituted only 6.34% and 15.87% respectively of all manually detected cells, thus creating a severe class imbalance that prevented our CNN from segmenting them. Contrastingly, automated structural measurement data (using large windows, same size as manual windows) performed better than manual structural data (by 0.131 for surgery and 0.194 for stimulation) simply due to the multiple orders of magnitude larger quantity of measurements (thousands vs 3–5) that were able to be collected and used to train the regressions. The predictive ability of our trained SVM might be greatly improved if rarer cell classes, related to damage, could be segmented on the same scales as normal cells by our CNN.

**Figure 1:**
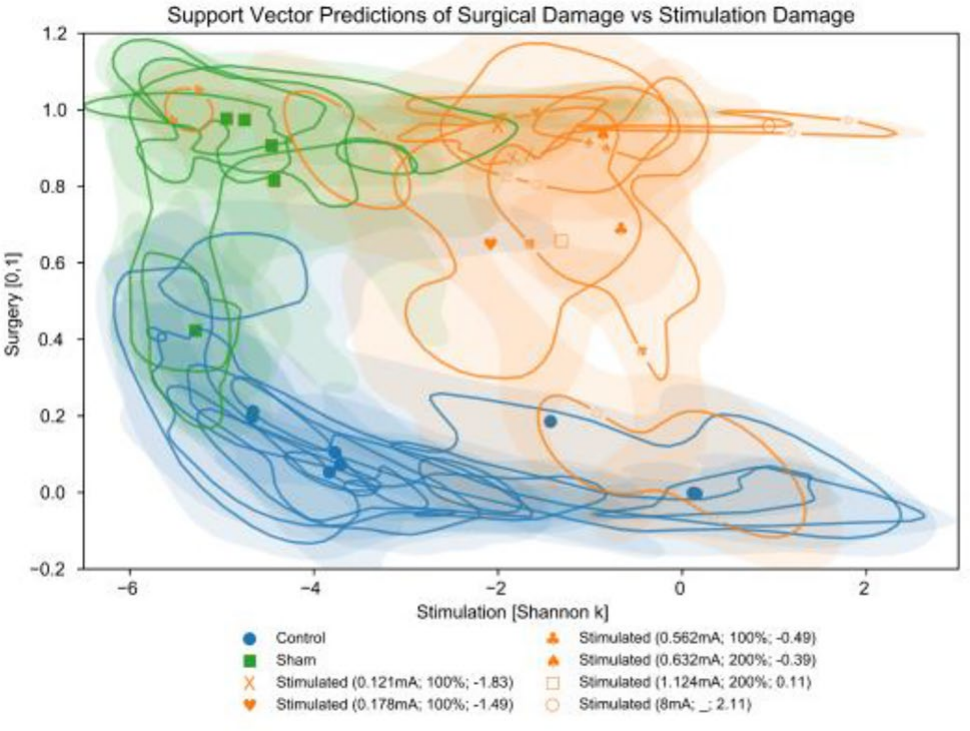
SVM regression values for each sample. For each sample, we applied the surgery and stimulation regressions that were trained using large-window pixel-wise data (fiber density, axon packing, and myelin packing). The average output was calculated for each sample with control samples in blue circles, sham samples in green squares, and stimulated samples in orange (with stimulation levels noted in mA; McCreery level; and Shannon k). Fast Fourier transform kernel density estimation was then used for each sample’s resulting 2D multivariate distribution to estimate the distribution’s 1 sigma (68% confidence interval) represented by the solid lines (stimulation samples lines also have associated marker) and each sample distribution’s 2 sigma (95% confidence interval) represented by the shaded regions. Some portions of the control and stimulated sample data (but none of the sham) were inferred to have changes related to high levels of stimulation but not related to the electrode being implanted (concentration of values in the bottom right of the plot). However, no stimulated sample data falls in the low surgery and low stimulation region (bottom left).

The surgery and Shannon *k* stimulation regressions, that were trained on automated large-window pixel-wise data, were applied to the entire sample data set and plotted in Fig. 1. While most control samples show the expected low levels of structural changes related to stimulation, the three control nerves with mean values and 68% confidence intervals above a Shannon k of -2 were taken from the legs contralateral to the three highest stimulated samples. This may suggest that contralateral sciatic nerves are not completely independent from effects of stimulation and a need for control samples taken from rats that haven’t undergone any electrode implantation procedure. Despite this, the surgery and stimulation regressions are detecting different changes in the histology as evidenced by the bottom-left of the plot (surgery < 0.3 & Shannon k < − 3.5) having control samples but no stimulated samples, and the top-right (surgery > 0.6 & Shannon k > − 1.5) having stimulated samples but no control samples (otherwise we would expect the 2D distributions to be colinear from the top-left to bottom-right as they measure the same differences from the sham samples). As shown by the sample markers for the stimulation samples, the 2D sample distributions can have multiple peaks, making the mean values (and other central tendencies) of the distributions less representative of the underlying data. The 1.124 mA stimulated sample has 3 peaks; one in the region of large surgery related changes and moderate stimulation changes (top-center), one in the region of small surgery related changes and large stimulation changes (bottom-right), and the last in the region of large changes from both sources (top-right). These multimodal distributions are due to changes from each source being localized to different regions of the nerves that can overlap; making where measurements are taken from the nerve much more important than if changes from both sources were uniform across the entire nerve.

## Discussion

The automated segmentation and feature extraction methods presented enable orders of magnitude more measurements to be made in a fraction of the time compared to traditional methods. Notably, our current CNN architecture limits data collection by not segmenting degenerated and hypomyelinated cells and could be improved by using an architecture capable of instance segmentation as well as handling the large class imbalances due to the additional cell categories. Our methods allow for measurement of localized changes to nerves; without more localized stimulation values, large window generalized metrics will be the best measurements of nerve differences related to stimulation. This study only used fixed diameter cuff electrodes on rat sciatic nerve and would benefit from replication with other electrode designs and geometries, in other peripheral nerves, and other animal models. Additional limitations are the use of only one stimulation protocol (stimulation waveform, duration of implantation/stimulation, recovery period between epochs and after stimulation) and the notable lack of a functional test to measure impact of observed histological changes. Despite these limitations, our results indicate that, although all but one stimulated sample are within expected safe levels of stimulation (Shannon *k* < 1), there are structural changes in the nerve that match the levels of electrical stimulation applied independently of the structural changes due to surgical implantation. And that these changes require non-Gaussian multivariate analysis techniques, such as ours, to be observed.

Knowing the extent a nerve changes and is damaged at different levels of stimulation (especially low levels traditionally thought to be safe) can aid in the development of more representative stimulation safety standards.

## Methods and procedures

### Electrical stimulation

The vertebrate animal experiments were done under the guidelines of the Public Health Service (PHS) Policy on Humane Care and Use of Laboratory Animals and the NIH Office of Laboratory Animal Welfare and reported in accordance with ARRIVE guidelines. The described procedures were approved by the University of Utah Animal Care and Use Committee. Adult Sprague Dawley rats (purchased in the 200–225 g range, used before reaching 300 g, 6 male and 5 female) were used for this study. Each rat underwent 3 phases in the experiment, implantation, stimulation, and euthanasia.

In the implantation phase, each rat was implanted with a fixed diameter multi-electrode cuff array (MECA, Microprobes for Life, Gaithersburg MD, USA) (see Supplementary Fig. S1 online) while under isoflurane anesthesia. The MECA was a standard nerve cuff with a 2 mm inner diameter, 3 contacts made from 100 µm diameter Pt wire with 1.5 mm spacing between contacts, and a 2 mm distance between each outer contact and the end of the cuff. The MECA’s connector was attached to the skull, the wire bundle was transcutaneously routed to the sciatic nerve, and the cuff was placed on and sutured to the sciatic nerve. The muscle and skin were sutured closed, and the rat recovered.

One week later, the rat underwent an electrical stimulation procedure under isoflurane anesthesia. The spinal neural Compound Action Potential (nCAP) was recorded with two needle electrodes (Horizon Subdermal Needle Electrodes, LifeSync Neuro, Coral Springs, FL) placed over the lumbar spine, with one electrode inserted into the skin overlying L5 vertebrae and second electrode inserted into the skin approximately 15 mm caudal to the first electrode. Recording ground was provided by a hypodermic needle inserted into the skin between these two electrodes and the location of the implanted cuff. The nCAP was measured as the difference between the two needle electrodes by a Cerebus System (Blackrock Microsystems, Salt Lake City, UT). The data were amplified, filtered, and recorded at 30,000 samples per second. The signals were filtered with a 3 Hz, first order, highpass filter and a 2000 Hz, fourth order lowpass Butterworth filter. Further, the Cerebus system recorded a digital pulse every time the stimulator started a stimulation pulse. The pulse was used to temporally align each nCAP response. The median nCAP response was calculated for a window 10 ms before to 10 ms after the pulse and the average A-alpha nCAP was quantified by extracting the peak-to-peak signal in a window between 2 and 5 ms after the pulse.

The two outer contacts of the MECA were attached to a stimulator (STG-4002-16 mA, Multichannel Systems, Baden-Württemberg, DE) that applied constant current, charge-balanced biphasic pulses with 100 µs per phase, and 400 µs inter-pulse period. The nCAP’s recruitment curve was acquired by stimulating at 5 Hz for 128 samples at current levels between 100 and 2000 µA, and the least current level that evokes the maximum A- nCAP response was extracted (referred to as the McCreery level). A current level was selected for the rest of the procedure that is: nearly zero (2 µA) to observe effects related to electrode implantation with negligible stimulation, a multiple of the McCreery level (100% or 200%) while still below Shannon’s current limit (k < 2) to observe expected safe but non-negligible levels of stimulation, or a user-selected ‘high’ current above Shannon’s current limits (k > 2) as reference for histological changes from expected unsafe stimulation. For sixteen 15-min epochs, the nerve was stimulated at the selected current level at 50 Hz (45,000 stimulation pulses per epoch). The epochs were separated by 20–30 s, during which the nerve was stimulated at 1 Hz to acquire the nCAP. After the stimulation was completed, the rat was recovered.

One week later, the rat was euthanized using exsanguination while unconscious (via deep isoflurane anesthesia), in compliance with the 2020 Edition of the AVMA Guideline for Euthanasia of Animals (Section M3.13.1). The rat was then perfused via cardiac puncture, starting with physiologically normal phosphate-buffered saline (PBS), followed by 4% formaldehyde and 2% glutaraldehyde in PBS. The region of the sciatic nerve with the MECA was excised, and a similar region from the contralateral (unimplanted) side. Both nerve samples were placed in a vial filled with 4% formaldehyde and 2% glutaraldehyde in PBS and stored in a 4C refrigerator for 1 week. They were transferred into a vial filled with 0.02% sodium azide in PBS and shipped for histological analysis.

### Experimental groups

Samples were categorized into three groups: 5 sham samples (electrode implanted, but negligibly stimulated [2 µA]), 6 stimulated samples (electrode implanted, and stimulated to some McCreery level), and 8 control samples (no electrode implanted) taken from the contralateral leg of the sham and stimulated samples (detailed in Supplementary Table S2 online).

### Histology

Samples were post-fixed with 1% osmium tetroxide for 2 h at 4 °C, incubated with 1% uranyl acetate solution for 1 h at 4 °C in the dark and dehydrated with gradual concentrations of ethanol (70%, 85%, 96%, 100%, 100%, 100%). The embedding was carried out using various proportions of propylene oxide and Epon mixture (3:1 for 4 h, 2:1 for 2 h and 1:1 overnight) (Electron Microscopy Sciences, Hatfield, PA), followed by pure Epon for a further night before the embedding in fresh Epon and placed at 60 °C for 48 h for complete hardening.

### Light microscopy

Samples embedded in Epon were sliced into semithin sections (500 nm). Slices from the location of the source electrode in the electrode cuff were stained with 1% toluidine blue and imaged with a light microscope (MoticEasyScan One™) at 80× magnification. After imaging, samples were inspected for fixation quality. Three control samples were unmeasureable due to issues with sample fixation (see Supplementary Fig. S3 online) resulting in considerably degraded image quality across the entire sample, causing them to be excluded from analysis. For most samples though, poor tissue fixation issues were limited to the very centers of the largest fascicles. These issues stem from the lipid-rich myelin sheathes acting as a barrier to fixative penetration^34^ and consequently effecting the higher density control samples more severely.

### Traditional measurements

Cell morphometrics and structural measures were calculated using ImageJ. The morphometric measurement process for each sample was to define the image resolution and free hand select the axon boundaries and fiber boundaries of 500 cells around the periphery of the nerve and 100–200 cells in the center. The lower cell sampling in the center was due to the previously mentioned issues with sample fixation at the center of some nerves. From the 2 contours, morphometrics could be calculated (axon/myelin/fiber area, axon/fiber equivalent diameter, axon/fiber mean diameter, axon/fiber aspect ratio, axon/fiber circularity, and g-ratio).

**Figure 2:**
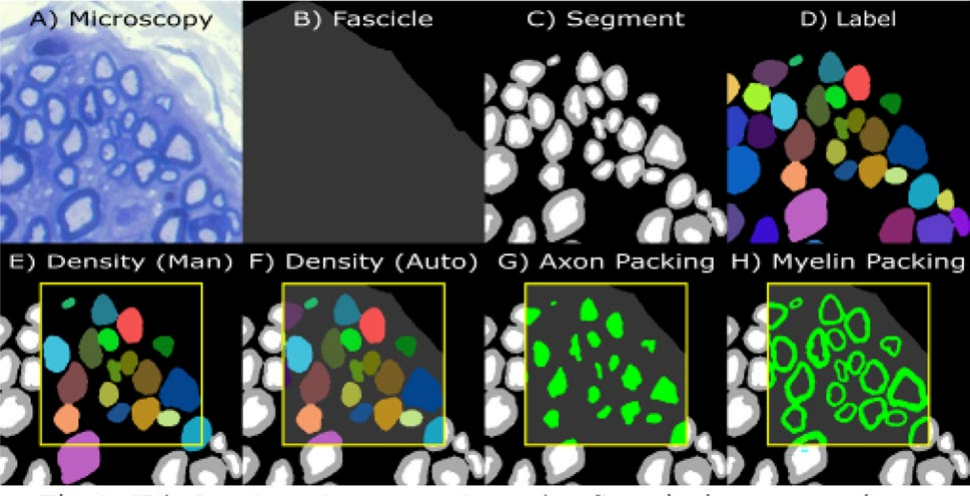
Window-based structural metrics. Steps in the automated process of instance segmentation of nerve fibers' axon and myelin (**A**–**D**). (**B**) Fascicles manually masked in dark gray with extrafascicular space and other exclusion regions in black. (**E**) Manually calculating fiber density (# fibers/window area) using the quadrat method to count fibers. (**F**) Fiber density measured by NeMeAn (# fibers/fascicle area) using fraction of fiber area in window to count fibers. Automation also enabled calculation of the packing (tissue area/fascicle area) of axons and myelin within the windows (**G** & **H** respectively).

The structural measurement process similarly starts by defining the image resolution. Then 3–6 windows (size: 200 × 200 µm, sometimes 150 × 150 µm if the sample did not admit larger squares) were selected randomly within the fascicles while avoiding blood vessels, blurred areas, and streaks. Within each window, the fibers were labeled and counted as either healthy, degenerated, or hypomyelinated. Cells touching the top or left window borders were excluded from these counts, according to the quadrat method as shown in Fig. 2E. Fiber density was then calculated for each of the 3 fiber types within each window. Due to the time and labor-intensive nature of manually segmenting cells, we were unable to get axon or myelin packing values.

Correlations in morphometric distributions in the nerve periphery were compared to measurements made in the center as shown in Supplementary Fig. S4. These outcomes indicated a positive and congruent relationship between the average axon diameter and the g-ratio in both the periphery and the center, thereby suggesting that measurements taken in the periphery of select sections are emblematic and yield pivotal insights into axon morphology within the peripheral nerve. While poor tissue fixation in some sections precluded the measurement of central axons due to preservation constraints, the data acquired from the periphery remain consistent and reliable for conducting our morphometric analysis employing sophisticated computational and artificial intelligence methodologies. This substantiates the judicious choice of prioritizing peripheral measurements as a valid and efficacious approach within the following computational methodologies.

### Automated high throughput measurements

**Figure 3:**
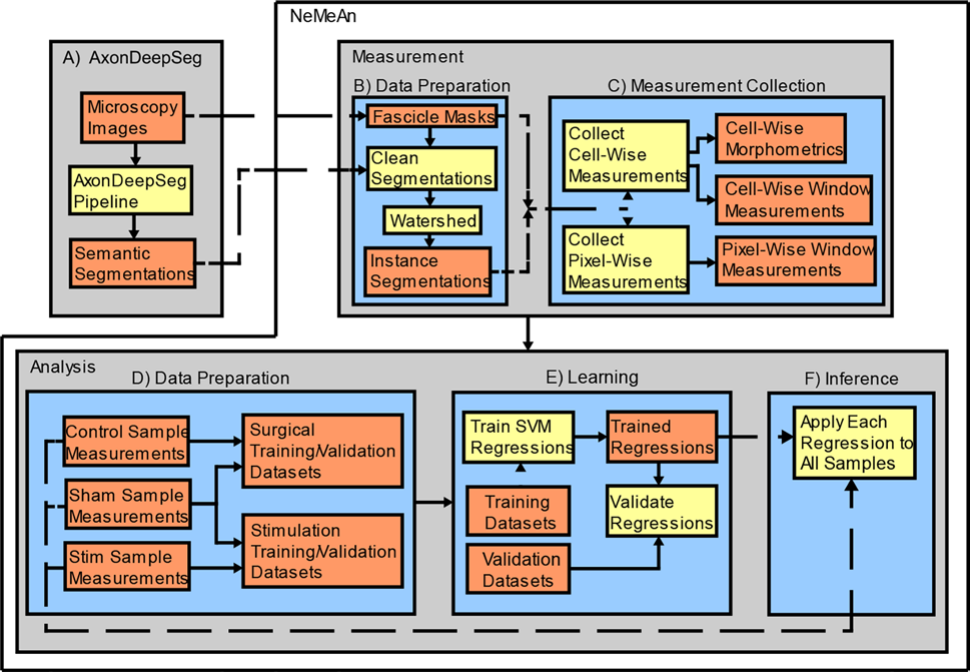
Overview of the NeMeAn data flow pipeline. AxonDeepSeg’s methods (**A**) were replicated to acquire semantic segmentations of myelin and axon from light microscopy images (resolution of 8 pixels/µm) stained with toluidine blue. To generate instance labels of myelinated fibers from semantic labels, the first data preparation step (**B**) clears erroneous semantic labels and then assigns myelin pixels to axons using the watershed algorithm. During the measurement collection step (**C**), each labeled myelinated fiber's morphometrics and window-based metrics are measured and collected into a “cell-wise” multivariate dataset. Separately in the same step (**C**), “pixel-wise” window-based metrics are collected for each possible window position in each sample. In the second data preparation step (**D**), multivariate measurements are split based on the sample type (control, sham, stim) and recombined into 2 datasets. Control and sham samples were used in the dataset for detecting changes related to surgical implantation of the cuff electrode. Sham and stimulated samples were used in the dataset for detecting changes related to stimulation level. These two datasets are then each split into training and validation sets. (**E**) A regression is fit to each of the surgery & stimulation training datasets and then evaluated on the data set aside for validation. Finally, both surgery & stimulation regressions are applied to all samples in the inference step (**F**).

The open-source convolutional neural network, AxonDeepSeg^19^, was developed at NeuroPoly to segment myelin and axon tissues in a range of imaging modalities using a U-net architecture trained on small data sets. We replicated their paper's CNN architecture and methods (Fig. 3A) (to apply this CNN to our light microscopy images (Fig. 2A) with the only changes being the training/validation data sets (due to our having light microscopy images instead of electron microscopy) and the patch sizes. We built training/validation data sets by manually segmenting myelinated fibers in non-overlapping patches from 8 images with a variety of tissue states and image quality conditions (patches sized 256 × 256px [0.125 µm/px]: 459 for training, 116 for validation). Labels for hypomyelinated axons and degenerated fibers were not included in the training/validation data due to it creating severe class imbalances (see “Results” Section).

Due to fixation issues with some tissue samples, some central nerve regions could not be segmented, neither manually, nor with the CNN. To address this, the fascicles were manually assessed for fixation quality and masked (Fig. 2B) to exclude these poor fixation regions. Also excluded from the fascicle masks were veins and streaks inside the fascicles (things normally avoided in manual measurements).

The trained network was then applied to our full set of sciatic nerve samples (1 image for each of the 19 samples). While the CNN was validated with an accuracy of 95% (F1/Dice Score: Axon:0.847, Myelin: 0.831), some post-processing was still necessary (Fig. 3B). The procedure consisted of removing axon/myelin labels that were inside the exclusion regions, removing cell labels with axon size less than 4 pixels (0.062 µm^2^) that were very rare [0.13% of cells] in manual measurements (and thus detections of this size by the CNN would likely be false positives from to noise in images), and removing any cells where axon is adjacent to background or myelin was not contiguous with an axon (as we are not measuring, and the CNN is not trained for, degenerated or hypomyelinated fibers).

AxonDeepSeg provides a semantic segmentation of axon and myelin (Fig. 2C), however for morphometric analysis, we need instance segmentations to perform measurements on individual fibers. Instance segmentations of axon are easy to acquire, however the myelin of adjacent cells is contiguous and require more work to separate. To separate these cells, we generated a height map where axon pixels are height 0, background pixels are height 1, and the height of myelin pixels are interpolated between them, as shown in Eq. (1). The watershed algorithm was then applied to this height map with labeled axons as seed regions, returning an axon label for each myelin pixel (Fig. 2D).

1 $${\text{myelin height}} = \frac{{\text{axon distance}}}{{{\text{axon distance}} + {\text{background distance}}}}$$

With the work of instance segmentation automated (Fig. 3B), we can quickly and consistently make a larger quantity and variety of measurements (Fig. 3C), like axon packing and myelin packing in addition to morphometrics and fiber density.

One concern for window-based metrics (fiber density & axon/myelin packing) was the effect of window size on the measurements. Using larger windows provides measurements that are more representative of the overall state of the fascicles; while smaller windows create a more localized measurement but can limit the max detectable distance between cells in extreme cases. We collected window-based automated measurements using two different window sizes (large 200 × 200 µm [700 × 700 px] matching window size used in manual measurements, and small 37.5 × 37.5 µm [200 × 200 px]).

These windowed measures are also affected by window placement. To understand the structural changes local to each cell and to combine with morphometric measurements into a multivariate data set, the windows can be selected such that there is one window centered on every cell (cell-wise measurements). The concern with this placement strategy is that when selecting windows, by centering each window on an axon, there will be an inherent survivorship bias skewing sampling towards fascicular regions with more cells and away from regions with lesioning. While much more computationally expensive, and unable to be combined with cell-wise morphometric data, it is possible to make the window-based structural measurements where each window is centered on each pixel of the fascicles (pixel-wise measurements resulting in making a measurement for every possible window placement within a fascicle). Using parallelization and data chunking^35–37^, the millions of pixel-wise measurements were able to be performed quickly. Both cell-wise and pixel-wise measurements were made for each sample.

When traditionally making window-based structural measurements (density & packing), the window area was used in the denominator. However, this limits where windows can be placed, since anytime the window encloses a region where fibers are not expected (such as along the edges of the fascicles), the measurement will be skewed downward. By using the area of the fascicle mask enclosed in the window instead of just window area (Fig. 2F–H), we were less restricted in the placement of our structural measurements. Another improvement enabled by automation stems from the way fibers were counted for fiber density. Since we know the total cross-sectional area of each fiber, we can count fibers that are cut by the window by the fraction of the fiber contained in the window instead of relying on the binary inclusion/exclusion of the quadrat method (Fig. 2F).

### Analysis of multivariate data

We wanted to determine if our multivariate data had trends following the sample's treatment (control, sham, or stimulation). To do so, we created 2 datasets (control-sham and sham-stimulation) (Fig. 3D) and attempted to fit a regression to each dataset using the support vector machine (SVM) algorithm from the scikit-learn python package^38^ and the function parameters listed in Table 2 (Fig. 3E). We used the Nystroem approximation of a radial basis function (RBF) kernel for the SVM regression model. The Nystroem approximation was selected because, while the RBF kernel is very powerful in fitting non-linear trends in non-Gaussian data, it becomes prohibitively slow for large data sets. In addition to using the Nystroem approximation to accelerate learning, we used stochastic gradient descent to train the SVM regressions on batches instead of the entire datasets).

**Table 2:** Support vector machine parameters.

RBF parameters	Values
γ	1/number of features
Number of components	100
Tolerance	1e-3
Regularization parameter C	1.0
ε	0.1
SGD Regressor Parameters	Values
Loss	ordinary least squares fit
Penalty (regularization term)	L2
α	0.0001
Fit intercept	True
Maximum iterations	200,000
Tolerance	1e−12
Shuffle between epochs	True
Learning rate	invscaling

The first regression was to identify trends related with surgical treatment (including the surgical procedure itself, how the electrode was fixed to the nerve, biocompatibility, and the prolonged presence of an implanted device). For this surgical regression, control samples were labeled as 0 and sham samples were labeled as 1. The regression was then fit using 70% of the multivariate measurements of the control and sham samples as the input training vectors and the sample labels as target values.

The second regression was to identify trends related with electrical stimulation and used sham and stimulated surgical samples labeled with their associated Shannon k level. The regression was then fit using 70% of the multivariate measurements of the sham and stimulation samples as the input training vectors and the sample labels as target values.

For validation, the coefficient of determination (R^2^ where a perfect score is 1; constantly predicting the average of the true output no matter the input results in a score of 0; and as regression gets worse the score decreases and can become arbitrarily negative) was calculated using the 30% of the sample data that was set aside during fitting.

These 2 regressions were trained and validated for several subsets of measurements (subsets referred to as training vectors and individual measurements as features). For manual and automated morphometric training vectors the 7 features used were axon/myelin cross-sectional areas, axon/fiber aspect ratios, axon/fiber circularities, and g-ratios. Automated windowed metric training vectors included 3 features from the same sized window (either small or large): fiber density, axon packing, and myelin packing. The R^2^ values from validation testing each regression are shown in Table 1.

The surgical and stimulation regressions for large-window pixel-wise metrics (which had the highest and second highest R^2^ for each target value respectively) were then applied to all samples (control, sham, and stim) to infer the distribution of samples related to each source of histological change. To visualize the 68% and 95% confidence intervals of these 2D distributions (Fig. 1), we used fast Fourier transform kernel density estimation (FFTKDE) with a bandwidth for each dimension selected using Scott’s Rule.

### Supplementary Information

Supplementary Information.

## Data Availability

The nerve measurement and analysis code is available as open source in GitHub (https://github.com/LazziLab/NeMeAn).

## References

[1] Johnson RL, Wilson CG (2018). A review of vagus nerve stimulation as a therapeutic intervention. J. Inflamm. Res..

[2] Toffa DH (2020). Learnings from 30 years of reported efficacy and safety of vagus nerve stimulation (VNS) for epilepsy treatment: A critical review. Seizure.

[3] Dai F (2020). Effects and mechanisms of vagal nerve stimulation on body weight in diet-induced obese rats. Obes. Surg..

[4] Grill WM, Kirsch RF (2000). Neuroprosthetic applications of electrical stimulation. Assist. Technol..

[5] Elefteriades JOHNA (2002). Long-term follow-up of pacing of the conditioned diaphragm in quadriplegia. Pacing Clin. Electrophysiol..

[6] Dhillon GS, Horch KW (2005). Direct neural sensory feedback and control of a prosthetic arm. IEEE Trans. Neural Syst. Rehabilit. Eng..

[7] Rossini PM (2010). Double nerve intraneural interface implant on a human amputee for robotic hand control. Clin. Neurophysiol..

[8] Clark, G. A. *et al.* Using multiple high-count electrode arrays in human median and ulnar nerves to restore sensorimotor function after previous transradial amputation of the hand. In *2014 36th Annual International Conference of the IEEE Engineering in Medicine and Biology Society* (2014).10.1109/embc.2014.6944001 10.1109/EMBC.2014.6944001 25570369

[9] Li AH (2022). Considerations in permanent implantation of peripheral nerve stimulation (PNS) for chronic neuropathic pain: An international cross-sectional survey of implanters. Pain Pract..

[10] Olson MD, Junna MR (2021). Hypoglossal nerve stimulation therapy for the treatment of obstructive sleep apnea. Neurotherapeutics.

[11] Wang M (2020). Percutaneous tibial nerve stimulation for overactive bladder syndrome: A systematic review and meta-analysis. Int. Urogynecol. J..

[12] Martin D (2021). Long-term results following electrical stimulation of the peroneal nerve using the ActiGait^®^ system in 33 patients with central drop foot. Innov. Surg. Sci..

[13] Günter C, Delbeke J, Ortiz-Catalan M (2019). Safety of long-term electrical peripheral nerve stimulation: Review of the state of the art. J. NeuroEng. Rehabilit..

[14] Merrill DR (2005). Electrical stimulation of excitable tissue: Design of efficacious and safe protocols. J. Neurosci. Methods.

[15] Cogan SF (2009). Sputtered iridium oxide films for neural stimulation electrodes. J. Biomed. Mater. Res.

[16] Cogan SF (2016). Tissue damage thresholds during therapeutic electrical stimulation. J. Neural Eng..

[17] Onken A (2021). Predicting corrosion delamination failure in active implantable medical devices: Analytical model and validation strategy. Bioengineering (Basel).

[18] Boehler C (2020). Tutorial: Guidelines for standardized performance tests for electrodes intended for neural interfaces and bioelectronics. Nat. Protoc..

[19] McCreery D (2010). Neuronal loss due to prolonged controlled-current stimulation with chronically implanted microelectrodes in the cat cerebral cortex. J. Neural Eng..

[20] Du, J. *et al.* Electrode spacing and current distribution in electrical stimulation of peripheral nerve: A computational modeling study using realistic nerve models. in *43rd Annual International Conference of the IEEE Engineering in Medicine and Biology Society*, (2021). 10.1109/EMBC46164.2021.9631068 PMC10072138 34892199

[21] Du J (2022). Electrical stimulation induced current distribution in peripheral nerves varies significantly with the extent of nerve damage: A computational study utilizing convolutional neural network and realistic nerve models. Lect. Note. Comput. Sci..

[22] Du J (2023). Electrical stimulation induced current distribution in peripheral nerves varies significantly with the extent of nerve damage: A computational study utilizing convolutional neural network and realistic nerve models. Int. J. Neural Syst..

[23] Shannon RV (1992). A model of safe levels for electrical stimulation. IEEE Trans. Biomed. Eng..

[24] McCreery DB, Agnew WF, Yuen TGH, Bullara L (1990). Charge density and charge per phase as cofactors in neural injury induced by electrical stimulation. IEEE Trans. Biomed. Eng..

[25] Agnew WF, McCreery DB (1990). Considerations for safety with chronically implanted nerve electrodes. Epilepsia.

[26] Christensen MB (2014). The foreign body response to the Utah slant electrode array in the cat sciatic nerve. Acta Biomater..

[27] Comin CH (2014). Statistical physics approach to quantifying differences in myelinated nerve fibers. Sci. Rep..

[28] Christensen MB, Tresco PA (2015). Differences exist in the left and right sciatic nerves of naïve rats and cats. Anat. Record.

[29] Christensen MB, Tresco PA (2018). The foreign body response and morphometric changes associated with mesh-style peripheral nerve cuffs. Acta Biomater..

[30] Meyerholz DK, Tintle NL, Beck AP (2018). Common pitfalls in analysis of tissue scores. Vet. Pathol..

[31] Zaimi A (2018). AXONDEEPSEG: Automatic axon and myelin segmentation from microscopy data using convolutional neural networks. Sci. Rep..

[32] Quitadamo LR (2017). Support vector machines to detect physiological patterns for EEG and EMG-based human–computer interaction: A review. J. Neural Eng..

[33] Balabin RM, Lomakina EI (2011). Support vector machine regression (LS-SVM): An alternative to artificial neural networks (ANNs) for the analysis of quantum chemistry data. Phys. Chem. Chem. Phys..

[34] Duncan ID, Radcliff AB (2016). Inherited and acquired disorders of myelin: The underlying myelin pathology. Exp. Neurol..

[35] Harris CR (2020). Array programming with NumPy. Nature.

[36] Miles, A. *et al*. zarr-developers/zarr-python: v2.4.0 (v2.4.0). Zenodo. (2020).

[37] Moritz, P. *et al*. Ray: A distributed framework for emerging AI applications. in *13th USENIX Symposium on Operating Systems Design and Implementation* (2018).

[38] Pedregosa F (2011). Scikit-learn: Machine learning in Python. J. Mach. Learn. Res..

