# Supplementary material for: Machine learning enables non-Gaussian investigation of changes to peripheral nerves related to electrical stimulation
Source: Sci Rep. 2024 Feb 2;14:2795. doi: 10.1038/s41598-024-53284-w (PMC10837107; doi:10.1038/s41598-024-53284-w)
Supplement: Supplementary file 1 — Supplementary Information. [file 41598_2024_53284_MOESM1_ESM.pdf]

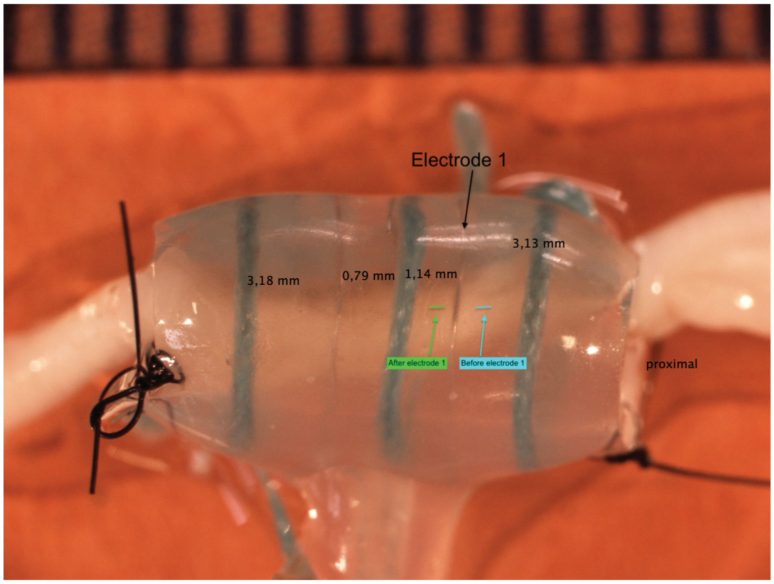

Supplementary Fig. S1. **Cuff electrode**. Fixed diameter multi-electrode cuff array (MECA, Microprobes for Life, Gaithersburg MD, USA) on a rat sciatic nerve. Histological sections taken from nerve at first electrode.

TABLE S2
RAT SCIATIC NERVE SAMPLES

Rat	Sex	Cuff Inner Diameter (mm)	Stimulus Voltage (mA)	McCreery Level (%)	Shannon k	Cells Detected	Pixels-Wise Windows	Contralateral Cells Detected	Contralateral Pixels-Wise Windows
Sham0	M	2	0.002	-	-5.39	6,057	23,042,278	7,603	26,874,578
Sham1	M	1	0.002	-	-5.09	6,352	28,831,907	Excluded due to poor fixation	
Sham2	F	2	0.002	-	-5.39	2,653	13,055,198	8,283	25,818,791
Sham3	F	2	0.002	-	-5.39	6,928	48,102,973	6,867	28,742,388
Sham4	F	2	0.002	-	-5.39	6,233	25,573,611	4,698	19,588,558
Stim0121	M	2	0.121	100	-1.83	8,347	73,450,640	Excluded due to poor fixation	
Stim0178	F	2	0.178	100	-1.49	5,403	28,171,487	5,200	23,687,179
Stim0562	M	2	0.562	100	-0.49	2,944	21,440,010	Excluded due to poor fixation	
Stim0632	M	2	0.632	200	-0.39	6,044	51,325,309	7,406	24,716,119
Stim1124	M	2	1.124	200	0.11	5,233	38,583,134	5,944	18,876,208
Stim8000	F	1	8	-	2.1	1,612	18,076,726	7,288	24,676,980

Sham experimental group had an electrode implanted, but negligibly stimulated. Stimulated experimental group had an electrode implanted and stimulated to various non-negligible levels with one sample stimulated above Shannon's current limit (k>2). Control samples were taken from the contralateral leg when not prevented by fixation issues.

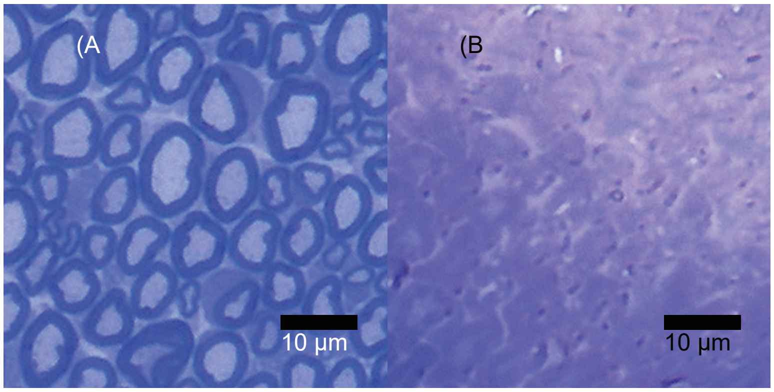

Supplementary Fig. S3. **Comparison of fixation quality.** (A) Example of good fixation in a semithin section of a control sample of rat sciatic nerve (embedded in Epon, stained with toluidine blue, and imaged with 80x light microscope). (B) Example of poor fixation from a control sample that prevented manual/automated segmentation and required its exclusion from the dataset.

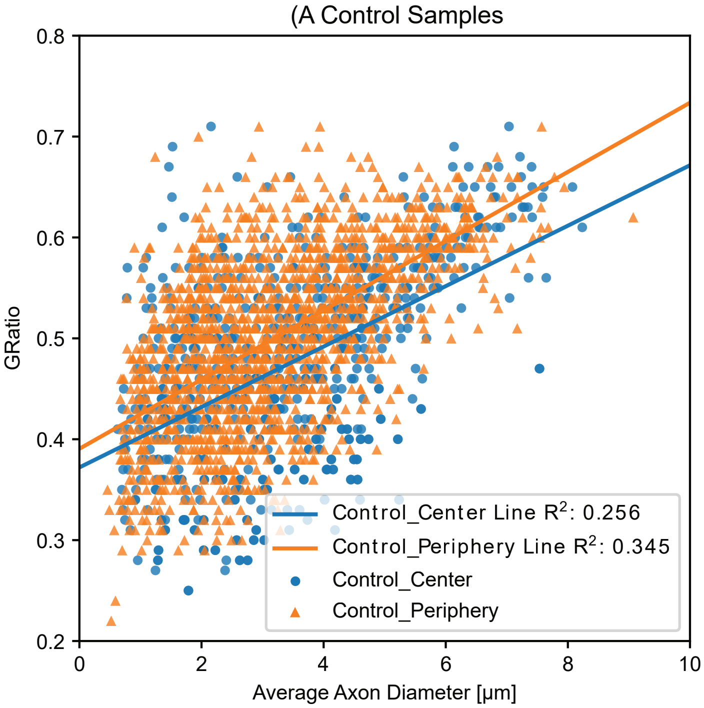

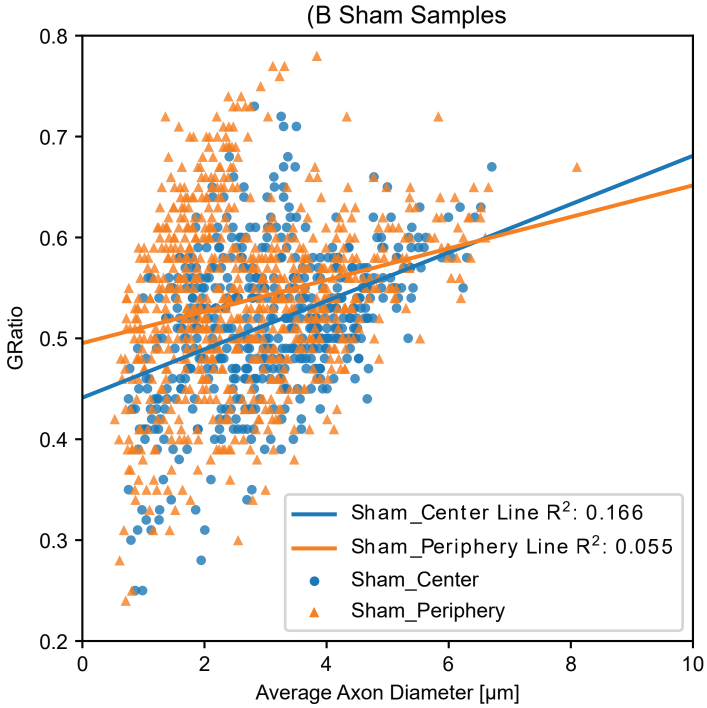

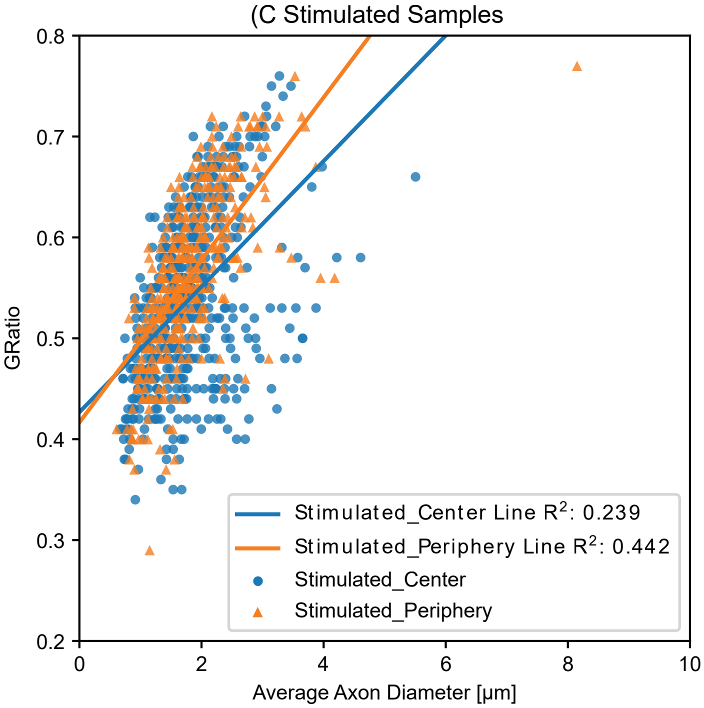

Supplementary Fig. S4. **Center & peripheral distributions**. 2D distributions of average axon diameter and g-ratio data as measured manually from central and peripheral regions of the rat sciatic nerve. The coefficients measure the linear relationship between the two measurement features. (A) Both regions (center and periphery) of control samples show similar correlations between average axon diameter and g-ratio. This suggest that the same trends are present in both regions of the nerve section. (B & C) In sham and stimulated nerve sections, we observed that the correlations, while slightly different in magnitude, remain positive, both between the periphery and the center and among different samples. This strongly suppoerts the idea that, despite the specific characteristics of stimulated and implanted nerves, peripheral measurements continue to be indicative of overall nerve morphology.
